# Patterns and Predictors of Depression Treatment among Older Adults with Parkinson's Disease and Depression in Ambulatory Care Settings in the United States

**DOI:** 10.1155/2018/3402983

**Published:** 2018-03-01

**Authors:** Sandipan Bhattacharjee, Nina Vadiei, Lisa Goldstone, Ziyad Alrabiah, Scott J. Sherman

**Affiliations:** ^1^Department of Pharmacy Practice and Science, College of Pharmacy, The University of Arizona, Tucson, AZ, USA; ^2^University of Southern California School of Pharmacy, Los Angeles, CA, USA; ^3^College of Pharmacy, King Saud University, Riyadh, Saudi Arabia; ^4^Department of Neurology, College of Medicine, The University of Arizona, Tucson, USA

## Abstract

Little is known regarding depression treatment patterns and predictors among older adults with comorbid Parkinson's disease and depression (dPD) in the United States (US). The objective of this study was to assess the patterns and predictors of depression treatment among older adults with dPD in the US. We adopted a cross-sectional study design by pooling multiple-year data (2005–2011) from the National Ambulatory Medical Care Survey (NAMCS) and the outpatient department of the National Hospital Ambulatory Medical Care Survey (NHAMCS). The final study sample consisted of visits by older adults with dPD. Depression treatment was defined as antidepressant use with or without psychotherapy. To identify predictors of depression treatment, multivariate logistic regression analysis was conducted adjusting for predisposing, enabling, and need factors. Individuals with dPD and polypharmacy were 74% more likely to receive depression treatment (odds ratio = 1.743, 95% CI 1.376–2.209), while dPD subjects with comorbid chronic conditions were 44% less likely (odds ratio = 0.559, 95% CI 0.396–0.790) to receive depression treatment. Approximately six out of ten older adults with PD and depression received depression treatment. Treatment options for dPD are underutilized in routine clinical practice, and further research should explore how overall medical complexity presents a barrier to depression treatment.

## 1. Introduction

Among neurodegenerative diseases, Parkinson's disease (PD) ranks second in frequency and importance [1]. Parkinson's disease is characterized by the well-studied motor symptoms: bradykinesia, tremor, rigidity, and postural imbalance [2]. Although less studied, the nonmotor symptoms are increasingly recognized as important targets of research due to their high prevalence and significant negative impact on the quality life of individuals with PD. Depression is one of the most common nonmotor symptoms of PD, with clinically significant depressive symptoms present in over one-third of individuals with PD [3]. It is thought that this is not only due to the psychosocial stress and disability experienced by individuals with PD, but also due to underlying neuroanatomical degeneration [4]. This includes neurodegenerative processes of the brainstem monoamine and indolamine afferents, along with various subcortical nuclei (ventral tegmental area, hypothalamus, dorsal raphe, and locus coeruleus) that have been implicated in depression [5]. Additionally research has shown that depression precedes the onset of motor symptom development and that dPD patients have been shown to be more depressed than those with other disabling medical illnesses [6, 7]. A relationship between severity of PD and depression has also been observed [8], and treatment of motor symptoms of PD has not been shown to consistently correlate with changes in mood [9, 10]. Therefore, depressive symptoms experienced in dPD may be attributed to the illness neurobiology, increasing the likelihood that depression treatment will eventually be needed during the course of illness. Depression in PD (dPD) not only significantly decreases quality of life [11] but is also associated with sleep disturbances, reduced functional status, and limitations of activities of daily living [12].

Antidepressant medication [13] and psychotherapeutic approaches, such as cognitive behavior therapy (CBT) [14], have shown to decrease depression scores and improve the quality of life among individuals with dPD. Unfortunately, the majority of individuals with dPD are untreated [15–17], and even in the case of treatment, up to 50% may still remain depressed, suggesting inadequate or ineffective treatment [18]. To the best of our knowledge, no study is available to date that examines the depression treatment pattern and predictors at the United States (US) national-level among older adults with dPD. Hence, we undertook this cross-sectional study to assess the patterns and predictors of depression treatment among older adults with dPD seeking care in ambulatory settings in the US.

## 2. Materials and Methods

### 2.1. Study Design

We adopted a cross-sectional study design by using multiple years (2005–2011) of data from the National Ambulatory Medical Care Survey (NAMCS) and the outpatient department (OPD) of the National Hospital Ambulatory Medical Care Survey (NHAMCS). Human subjects review was not required for this study according to The University of Arizona Institutional Review Board.

### 2.2. Data Source

Nationally representative information related to ambulatory medical care services use and provisions in nonfederally employed physician offices and outpatient departments of noninstitutional general and short-stay hospitals are captured by NAMCS and NHAMCS, respectively. NAMCS and NHAMCS are ongoing yearly surveys administered by the National Center for Health Statistics (NCHS) of the Centers for Disease Control and Prevention (CDC) [19]. National-level estimates are obtained by using the weight assigned to each visit.

A visit, which serves as the basic sampling unit for NAMCS and NHAMCS, is defined as a direct, personal encounter between a physician or a staff member working under a physician's direction for the purpose of obtaining care and providing health services. A multistage probability design is used by NAMCS for data collection, where the initial stage involves drawing probability sample from primary sampling units (PSUs) such as counties, groups of counties, county equivalents, or towns and townships. Subsequent to this stage, a probability sample of practicing physicians from each of the PSUs is obtained. In the final stage, which involves a two-step process, patient visits from the yearly practices of sampled physicians are selected. In the first step, the whole physician sample is split into 52 random subsamples of approximately identical size, and each subsample is randomly assigned to one of the 52 weeks during the survey year. In the second step, during the assigned week a systematic random sample of visits are selected by the physicians. Using the Patient Record Form (PRF), which is the data collection form for NAMCS and NHAMCS, a wide array of information with respect to patient characteristics, physician characteristics, diagnoses, medications prescribed, and the delivery of therapeutic services are collected by NAMCS. NAMCS and NHAMCS can record up to three diagnoses codes and eight prescription medications for each visit.

Data collection is conducted using a multistage probability sample survey involving selection of probability samples of PSUs, hospitals from each PSU, some or all outpatient and emergency departments from hospitals, and patient visits within these departments. The final stage of sampling in the NHAMCS is similar to that of the NAMCS. Due to the similarity of the medical care provided in OPD and office-based settings, only the OPD portion of the NHAMCS was used for this study. Data collection of NHAMCS was conducted using a similar PRF as the NAMCS.

### 2.3. Study Population

The final study sample consisted of visits by older adults (age ≥ 65 years) with PD and depression. PD was identified by using International Classification of Diseases, Ninth Revision, Clinical Modification (ICD-9-CM) of 332.xx [20]. Depression diagnosis was identified if answer to the question “Regardless of the diagnoses written ... does the patient now have: depression?” was “yes” [21]. To supplement chronic conditions, this item was added since 2005, and the robustness of this item has been described elsewhere.

### 2.4. Dependent Variable

Depression treatment, which was the dependent variable for this study, was defined as antidepressant use with or without psychotherapy. Antidepressant use was determined using generic drug codes and Multum Lexicon Codes. Due to small sample size, antidepressants were classified into the selective serotonin reuptake inhibitors (SSRIs) and other antidepressants. Psychotherapy was ascertained from the variable (PSYCHOTH) available in NAMCS and NHAMCS.

### 2.5. Independent Variables

The Anderson Behavioral Model (ABM) was used as the conceptual framework for this study, and the independent variables were classified as (i) predisposing, (ii) enabling, and (iii) need factors [22]. Predisposing factors comprised of age (65–74 years and ≥75 years), gender (male/female), race/ethnicity (white only non-Hispanic and others), geographical region (south and others), and metropolitan status (metropolitan and nonmetropolitan). Enabling factors consisted of health insurance status (government-Medicaid/Medicare and others), new patient visit (yes/no), and physician/clinic specialty (general and family practice and others). Need factors constituted of receipt of new prescription during the visit (yes/no), total number of chronic conditions, and total number of medications used.

### 2.6. Statistical Analysis

Ambulatory visits at national-level were reported in terms of weighted frequencies (in millions) and weighted percentages. To identify the predictors of depression treatment, multivariate logistic regression analysis was conducted adjusting for predisposing, enabling, and need factors. Survey procedures (SURVEYFREQ, SURVEYMEANS, and SURVEYLOGISTIC) were used to adjust for the complex survey design of NAMCS-NHAMCS to obtain national-level estimates in SAS version 9.4 (SAS Institute Inc., Cary, NC, USA).

## 3. Results

According to NAMCS-NHAMCS 2005–2011, approximately 9.3 million ambulatory visits (national estimation) recorded PD diagnosis, among which approximately 1.7 million visits (18.18%, 95% confidence interval (CI): 13.03%–23.33%) recorded a concurrent depression diagnosis forming the final study sample.

Individual-level characteristics of the study sample in terms of predisposing, enabling, and need factors are provided in Table 1 as weighted frequencies of visits in millions (national level) and their corresponding weighted percentages. Majority of the visits by the study sample consisted of individuals aged 75 years and older (55.29%), men (54.45%), and whites (non-Hispanic) (87.03%), who resided in metropolitan areas (79.07%), had some form of government insurance (85.04%) and were from Southern US (48.59%). An overwhelming majority (86.59%) of the study sample visits were recorded in physician/clinic specialties other than general and family practice and involved patients already established with the physician/clinic (87.16%). Mean total number of medications used and total chronic conditions recoded was 4.79 (S.E. 0.36) and 3.20 (S.E. 0.28), respectively (data not presented in tabular form), in the study sample.

Depression treatment (antidepressant with or without psychotherapy) was recorded in 57.63% (95% CI: 44.30%–70.97%) of the study sample visits. Among antidepressants, SSRIs were the most prescribed class, accounting for approximately 70% of antidepressant use (69.57%, 95% CI: 49.36%–89.78%). We were not able to estimate the national-level percentage of older adults with dPD receiving psychotherapy due to small sample size (*N*=5).

Table 2 summarizes findings from the multivariate logistic regression analyses to ascertain the predictors of depression treatment. Men were 64% less likely (adjusted odds ratio (AOR): 0.36, 95% CI: 0.14–0.93) than women to receive depression treatment. Individuals with dPD and polypharmacy were 74% more likely to receive depression treatment (odds ratio = 1.743, 95% CI 1.376–2.209), while dPD subjects with comorbid chronic conditions were 44% less likely (odds ratio = 0.559, 95% CI 0.396–0.790) to receive depression treatment.

## 4. Discussion

To the best of our knowledge, this is the first study to assess the depression treatment patterns and predictors among older adults with dPD at the national level in the US ambulatory care settings. Presence of depression has been associated with death or suicidal ideation [23], and hence, understanding depression treatment patterns in this vulnerable population is critical. Our study findings suggest that approximately six out of ten older adults with dPD received some form of depression treatment during their ambulatory visits. This estimate is higher compared to previous studies examining depression treatment patterns among individuals with dPD [18, 24]. A study by Weintraub et al. [18] using a convenience sample of patients at a PD center observed that among individuals with PD who met depressive disorder criteria, only one-third of them received antidepressant treatment. The study by Bega et al. [24] used the National Parkinson Foundation-Quality Improvement Initiative (NPF-QII) data and found that among individuals with PD meeting the depression diagnosis cut-off, only 33% used antidepressants, 6% used health services, and 14% used combination of antidepressants and health services. The convenience sample used by Weintraub et al. [18] or the NPF-QII data [24] are not nationally representative sample of US and as such lacks generalizability. Moreover, the data from Weintraub et al. [18] is more than a decade old. It is possible that the depression treatment patterns have changed since this published study. Although it is difficult to make direct comparisons between our study sample and these published studies, our findings show higher proportions of depression treatment.

Findings from our study indicate that SSRIs were the most prescribed antidepressant class (representing 70% of the overall antidepressant use) among older adults with dPD. This estimate is similar to the Weintraub et al. [18] study, which observed 69.6% of antidepressant use to be accounted by SSRIs. Despite the high use of SSRIs to treat depression among individuals with PD, a recent network meta-analysis [25] demonstrated that evidence to support efficacy and acceptability of SSRIs to treat depression in PD is insufficient and concluded that SSRIs may actually be the last treatment choice in these patients. Hence, findings from our study have implications for current practice. Tricyclic antidepressants can alternatively be considered given they have demonstrated ability to delay the need for dopaminergic therapy in early PD [26]. However, while anticholinergic medications such as tricyclic antidepressants can improve movement problems in PD, they carry the risk of adverse mental effects such as confusion, memory problems, hallucinations, and restlessness [27, 28]. In an existing literature review [29], it was summarized that while case reports have suggested SSRIs may worsen motor symptoms, these disturbances are reversible and generally not severe. A prior prospective study found no difference in serious extrapyramidal symptoms with the use of antidepressants versus dopaminergic drugs in dPD [30], and it is reported the potential benefit of SSRIs in dPD outweigh the risk [31]. Further studies are still needed to clarify whether SSRIs represent the best therapeutic index among available classes of agents.

Our study observed that males were less likely to receive depression treatment compared to females. Although studies specific to ambulatory settings are lacking, this finding is consistent with a study by Fernandez et al. [32] among nursing home PD residents, which observed that irrespective of behavioral symptoms, females with PD were more likely to receive antidepressants compared to males. This can partly be attributed to the fact that women with PD exhibit higher depression rates compared to men [33]. Our finding is also consistent with that of the general population that shows women to be two-and-half times more likely to receive antidepressants compared to men [34]. Piecing together findings from our study and existing studies, we can speculate presence of gender differences in terms of depression and antidepressant use among individuals with PD, and future research is warranted to elucidate and address this difference.

Our study findings indicate that the chances of receiving antidepressants were positively correlated with total number of medications recorded at the sampled visit. This may reflect a higher likelihood of prescribing antidepressants to PD patients with more advanced disease progression. It should be noted that this does not necessarily signify dPD patients having a delay in treatment, since psychotherapy is sometimes used in place of medication to avoid polypharmacy. Polypharmacy may lead to higher risk of adverse events such as fall frequency, fall severity, hyponatremia, bleeding, and drug-drug interactions [35]. Hence, healthcare providers should be aware of the overall medication burden and specific drug interactions when prescribing SSRIs for dPD. Finally, we observed that there was a lower likelihood of receiving depression treatment with increase in number of chronic diseases. This finding can be partially explained by competing demands arising from presence of other chronic conditions [36], having to rule out depression due to underlying untreated/unresolved medical conditions, as well as presence of drug-drug interactions [37].

The following limitations should be kept in mind while interpreting these findings. There is a possibility of statistical under power as the final unweighted study sample (*N*=133) was small. Chances of underestimation of disease conditions are possible as NAMCS and NHAMCS provide up to only three diagnoses per visit. Our study did not adjust for sedation, pain, sleep, and appetite stimulation for which antidepressants are also prescribed. Existing literature suggests that the ICD-9-CM code of 332.0 is often used to identify other conditions such as atypical parkinsonism, drug-induced parkinsonism, and idiopathic PD, and this code is unable to differentiate between parkinsonism and PD [38]. Hence, another limitation of this study is that the use of the 332.xx code may not be able to distinguish between PD and other forms of parkinsonism. We did not have information related to duration and severity of PD and depression, antidepressant dose, activities of daily living, instrumental activities of daily living, and functional status. Patient and physician preferences were also not available in the dataset. Furthermore, to achieve appropriate relative standard error, several variable categories (such as antidepressant classes) had to be combined to achieve reliable estimates, and we were not able to estimate the national-level estimate for psychotherapy use. Some other limitations include the possibility for reporting errors and coding errors, and interviewer effects should also be considered. Causal inferences cannot be reached due to the cross-sectional study design. Finally, we were able to use up to 2011 data, as the latest publically available OPD NHAMCS data is up to 2011.

## 5. Conclusion

Approximately six out of ten older adults in the US with PD and depression received depression treatment. SSRIs were most frequently prescribed, and gender, number of medications prescribed during visit, and number of chronic conditions were significantly associated with depression treatment among older adults with dPD. Psychotherapy is underutilized in this study sample. Future real-world long-term studies should investigate health outcomes associated with depression treatment in this vulnerable population.

## Figures and Tables

**Table 1:** Demographic and clinical characteristics and depression treatment of older adults with Parkinson disease and depression.

Characteristics	Wt. Freq. (millions)	Wt. %
Predisposing factors		

Age		
65–74	0.756	44.70
≥75	0.935	55.30
Gender		
Male	0.921	54.45
Female	0.770	45.55
Race/ethnicity		
White only, NH	1.472	87.03
Others	0.219	12.97
Geo region		
West	0.288	17.05
Northeast	0.384	22.72
Midwest	0.197	11.64
South	0.822	48.59
Metro status		
Metro	1.337	79.07
Nonmetro	0.354	20.93

Enabling factors		

Insurance		
Govt. insurance	1.438	85.04
Others	0.253	14.96
Physician/clinic specialty		
General and family practice	0.227	13.41
Others	1.464	86.59

Need factors		

New prescription during visit		
≥1	0.723	42.74
No	0.968	57.26
New patient		
Yes	0.217	12.84
No	1.474	87.16
Anti-Parkinson medication		
Yes	0.750	44.36
No	0.941	55.64
Chronic diseases		
Arthritis	0.411	24.31
Asthma	0.130	7.71
Cancer	0.084	4.97
CHF	0.556	3.29
COPD	0.194	11.49
Diabetes	0.624	36.91
HYPLIPID	0.649	38.36
HTN	0.832	49.17
IHD	0.338	19.96
CEBVD	0.199	11.78
Osteoporosis	0.088	5.19

Overall depression treatment		

Depression treatment	0.975	57.63

*Note.* Based on unweighted *N*=133 (nationally representative weighted *N*=1.7 million) ambulatory visits of older adults (age ≥ 65 years) with Parkinson's disease and depression using NAMCS and NHAMCS 2005–2011 data; NAMCS: National Ambulatory Medical Care Survey; NHAMCS: National Hospital Ambulatory Medical Care Survey; Wt: weighted; Freq.: frequency; NH: non-Hispanic; Govt.: government; CHF: congestive heart failure; COPD: chronic obstructive pulmonary disease; HYPLIPID: hyperlipidemia; HTN: hypertension; IHD: ischemic heart disease; CEBVD: cerebrovascular disease.

**Table 2:** Predictors of depression treatment among older adults with Parkinson disease and depression.

Characteristics	Odds ratio	95% CI	Significance
Predisposing factors			

Age			
65–74	Ref.		
≥75	1.008	0.24, 4.26	0.9909
Gender^∗^			
Female	Ref.		
Male	0.359	0.14, 0.93	0.0361
Race/ethnicity			
White only, NH	1.044	0.18, 6.1	0.9611
Other	Ref.		
Geographic region			
South	0.519	0.23, 1.15	0.1025
Other	Ref.		
Metro			
Metro	Ref.		
Nonmetro	0.584	0.11, 3.22	0.5276

Enabling factors			

Physician/clinical specialty			
General and family practice	Ref.		
Others	2.943	0.57, 15.16	0.1903
Insurance			
Govt. insurance	Ref.		
Others	0.776	0.23, 2.63	0.6755

Need factors			

New prescription			
No	Ref.		
≥1	3.019	0.99, 9.12	0.0501
Patient established			
New	Ref.		
Yes	5.43	0.66, 44.97	0.1134
Number of medications^∗^	1.743	1.38, 2.21	<0.0001
Number of chronic conditions^∗^	0.559	0.39, 0.79	0.0016

*Note.* Based on unweighted *N*=133 (nationally representative weighted *N*=1.7 million) ambulatory visits of older adults (age ≥ 65 years) with Parkinson's disease and depression using NAMCS and NHAMCS 2005–2011 data; NAMCS: National Ambulatory Medical Care Survey; NHAMCS: National Hospital Ambulatory Medical Care Survey; Ref.: reference group; AOR: adjusted odds ratio; CI: confidence interval; NH: non-Hispanic; NUMMED: total number of medications; TOTCHRON: total number of chronic conditions; ^∗^statistically significant at *p* < 0.05.
